# A Rare Case of Autoimmune Hepatitis-Primary Biliary Cholangitis Overlap Syndrome in a Male Patient

**DOI:** 10.7759/cureus.77023

**Published:** 2025-01-06

**Authors:** Bushra Amer, Waleed Khozaigi, Latifah D Hawshab, Fatimah Hawshab, Khaled Khozaigi, Khaled M Darwesh

**Affiliations:** 1 Department of Internal Medicine, Berkshire Medical Center, Pittsfield, USA; 2 Department of Gastroenterology, Prince Mohammed Bin Abdulaziz Hospital, Riyadh, SAU; 3 Department of Gastroenterology and Hepatology, Ahmad Mahir Hospital, Cairo, EGY; 4 Department of Gastroenterology and Hepatology, 6th of October Hospital, Cairo, EGY; 5 College of Medicine, Alfaisal University, Riyadh, SAU; 6 Department of Internal Medicine, Providence St. Peter Hospital, Olympia, USA

**Keywords:** autoimmune hepatitis, overlap syndrome, paris criteria, primary biliary cholangitis, rare case

## Abstract

Autoimmune liver diseases, such as autoimmune hepatitis (AIH) and primary biliary cholangitis (PBC), present significant diagnostic and therapeutic challenges due to overlapping features and potential for severe complications. AIH-PBC overlap syndrome, a rare condition, combines characteristics of both diseases but lacks standardized treatment protocols. We present the case of a 42-year-old male with elevated liver function tests, pruritus, flatulence, and epigastric pain. Laboratory findings revealed a cholestatic liver function pattern, a highly positive antinuclear antibody titer, weakly positive anti-smooth muscle antibody, elevated immunoglobulin G, and negative viral and anti-mitochondrial antibody markers. Diagnostic imaging, including abdominal ultrasound and magnetic resonance cholangiopancreatography, demonstrated mild fatty liver, slight irregularity in the left biliary duct wall, and a normal common bile duct without significant abnormalities. Liver biopsy confirmed chronic hepatitis with dense portal lymphoplasmacytic infiltrate, scattered eosinophils, moderate interface hepatitis, and mild lobular necroinflammation, consistent with AIH-PBC overlap syndrome. The Paris criteria were used to establish the diagnosis. Treatment with ursodeoxycholic acid, prednisolone, and azathioprine resulted in significant clinical and biochemical improvement.

## Introduction

Autoimmune liver diseases pose significant diagnostic and therapeutic challenges due to their complexity and potential for severe complications. Autoimmune hepatitis (AIH) is a chronic immune-mediated inflammatory liver disease that can affect people of all ages and ethnicities, with a higher prevalence in females. Its diagnosis is based on a combination of clinical features, laboratory test results, and liver histology [1-4]. The International Autoimmune Hepatitis Group (IAIHG) has established two reliable scoring systems: the revised system introduced in 1999 and the simplified version released in 2008. Both are endorsed by the clinical practice guidelines of the European Association for the Study of the Liver and the American Association for the Study of Liver Diseases [1,5-6]. Primary biliary cholangitis (PBC) is an uncommon autoimmune disorder that predominantly targets the bile ducts within the liver. This condition leads to inflammation and scarring of the biliary ducts, resulting in cholestasis and potentially progressing to liver cirrhosis over time. PBC predominantly affects women, with an incidence approximately nine times higher in females than in males. It most commonly occurs in individuals aged 40 years or older [7]. Overlap syndromes, such as AIH-PBC, are rare but clinically significant autoimmune liver diseases, occurring in 1-3% of patients with PBC and 7% of those with AIH. These syndromes are diagnosed using the "Paris criteria," which require at least two key diagnostic features from each condition. For PBC, the criteria include an alkaline phosphatase (ALP) level greater than twice the upper limit of normal (ULN), gamma-glutamyl transpeptidase (GGT) levels more than five times the ULN, the presence of anti-mitochondrial antibodies (AMAs), and histological evidence of florid bile duct lesions. For AIH, the diagnostic criteria involve alanine aminotransferase (ALT) levels exceeding five times the ULN, serum IgG levels at least twice the ULN, or the presence of smooth muscle antibodies (SMAs) [1,8]. Due to the rarity and complexity of AIH-PBC overlap syndrome, standardized treatment protocols are lacking. Most studies recommend a regimen combining ursodeoxycholic acid and corticosteroids, with or without azathioprine, which often yields promising results. Some patients may also benefit from ursodeoxycholic acid alone or corticosteroids with or without azathioprine. For those intolerant to or unresponsive to corticosteroids and/or azathioprine, second-line therapies such as tacrolimus, mycophenolate mofetil, or cyclosporine have shown some success [8]. This complexity is highlighted by the case of a 42-year-old male who presented to the gastroenterology clinic with elevated liver function tests (LFTs), pruritus, flatulence, and epigastric pain. The patient responded positively to a combination treatment of ursodeoxycholic acid, prednisolone, and azathioprine.

## Case presentation

A 42-year-old male presented to the gastroenterology department with elevated LFTs, pruritus, flatulence, and epigastric pain, which was occasionally relieved by proton pump inhibitors. He reported no altered bowel habits, jaundice, or changes in urine or stool color. His medical history included mild depression. He denied any use of alcohol, tobacco, or illicit drugs, as well as recent travel or a significant family history of liver disease. On examination, excoriation marks were noted on his abdomen and lower limbs, but there were no signs of jaundice, hepatomegaly, or stigmata of chronic liver disease. Initial laboratory tests revealed elevated liver enzymes, including aspartate transaminase (AST) at 70 U/L, ALT at 270 U/L, ALP at 475 U/L, and total bilirubin at 1.7 mg/dL. Repeat testing showed a cholestatic LFT pattern, with GGT at 1707 U/L, ALP at 709 U/L, total bilirubin at 2.1 mg/dL, AST at 124 U/L, and ALT at 380 U/L (Table 1).

**Table 1:** Laboratory tests

Analyte	Patient value	Reference range
Hemoglobin (g/dL)	13	13-17
Leukocytes/μL	5,000	4,000-11,000
Platelets/μL	240,000	150,000-400,000
International normalized ratio	1	0.8–1.2
Creatinine (mg/dL)	0.7	0.7–1.3
Albumin (g/dL)	4.1	3.4–5.4
Aspartate aminotransferase (U/L)	124	<34
Alanine aminotransferase (U/L)	380	<35
Gamma-glutamyl transpeptidase (U/L)	1,707	12-64
Alkaline phosphatase (U/L)	709	13-43
Total bilirubin (mg/dL)	2.1	0.1–1.2
Immunoglobulin G (mg/dL)	3,700	650-1,600
Antinuclear antibody	1:640	<1:80
Anti-smooth muscle antibody	1:48	<1:40
Anti-mitochondrial antibodies	Negative	
Anti-liver kidney microsomal antibodies	Negative	
Hepatitis B surface antigen	Negative	
Hepatitis C antibody	Negative	

**Figure 1:**
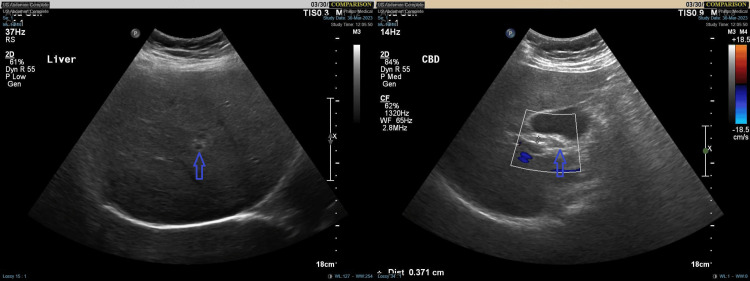
Abdominal ultrasound shows mild fatty liver, minimal gallbladder sludge without cholecystitis, normal common bile duct and portal vein, and no spleen abnormalities or free fluid.

Abdominal ultrasound findings revealed the liver with mildly increased parenchymal echogenicity, consistent with mild fatty liver, but no focal lesions or intrahepatic duct dilatation. Minimal gallbladder sludge was observed without evidence of acute cholecystitis. The common bile duct (CBD) was normal, and the portal vein was patent. The spleen was of normal size and echogenicity without focal lesions, and no free fluid was detected (Figure 1).

**Figure 2:**
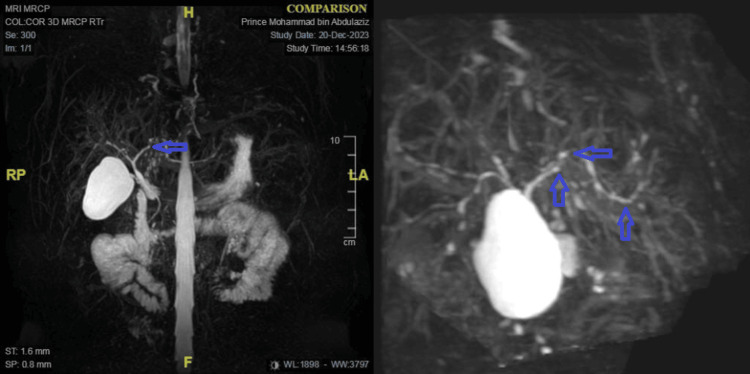
Magnetic resonance cholangiopancreatography shows slight irregularity in the left biliary duct wall without dominant stricture or intrahepatic duct dilatation. The common bile duct is normal in diameter, with unremarkable liver and gallbladder findings.

Magnetic resonance cholangiopancreatography findings revealed slight irregularity in the wall of the left biliary duct but no dominant stricture or intrahepatic biliary duct dilatation. The CBD diameter was within normal limits, and the liver and gallbladder appeared normal (Figure 2).

**Figure 3:**
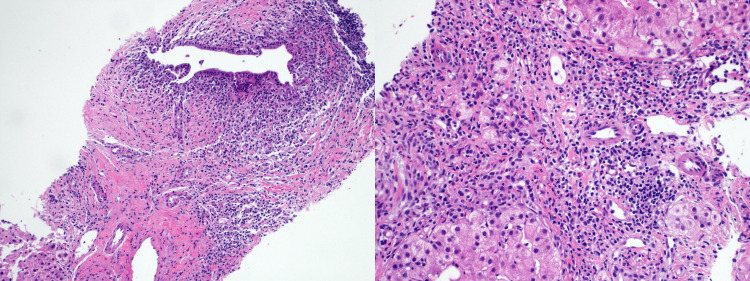
The liver biopsy revealed chronic hepatitis characterized by a dense portal lymphoplasmacytic infiltrate, scattered eosinophils, moderate interface hepatitis, and mild lobular necroinflammation.

Esophagogastroduodenoscopy and colonoscopy were unremarkable. Given the persistent elevation in liver enzymes and the clinical presentation, further diagnostic evaluation was conducted to investigate the presence of an underlying autoimmune liver disease. Autoimmune markers revealed a highly positive antinuclear antibody (ANA) titer of 1:640, weakly positive anti-SMA, negative AMA, and elevated immunoglobulin G (IgG) at 3,700 mg/dL. Viral markers were negative (Table 1). A liver biopsy confirmed chronic hepatitis, featuring a dense portal lymphoplasmacytic infiltrate, scattered eosinophils, moderate interface hepatitis, and mild lobular necroinflammation (Figure 3), findings consistent with AIH-PBC overlap syndrome.

The patient was initiated on a treatment regimen of ursodeoxycholic acid, prednisolone, and azathioprine, which led to marked clinical and biochemical improvement.

## Discussion

AIH and PBC are distinct autoimmune liver diseases that primarily affect women. Both conditions are characterized by dysfunctional T-cell activity, resulting in autoimmune-mediated destruction of liver architecture [9]. AIH is characterized by inflammation and hepatocyte injury, while PBC primarily causes biliary damage and cholestasis. In classical AIH, minimal biliary damage may occur, whereas minimal interface hepatitis can be seen in histological biopsies of PBC patients [10]. However, a spectrum exists between these diseases, with varying degrees of cholangitis, interface hepatitis, and lobular hepatitis. When both biochemical and histological features of AIH and PBC are distinctly present, the condition is classified as AIH-PBC overlap syndrome [10]. While the exact mechanism behind the lower incidence of these conditions in males remains unclear, factors such as sex hormones and epigenetics likely play a role. Most available data are derived from female subjects, with few studies examining male patients with overlap syndrome [9]. The diagnosis of AIH-PBC overlap syndrome is most commonly made using the Paris criteria, as recognized by international guidelines. In our case, the diagnosis was established based on these criteria. For PBC, the patient fulfilled two criteria: elevated cholestatic enzymes, with ALP levels of 709 U/L (more than twice the ULN) and GGT levels of 1707 U/L (exceeding five times the ULN), along with histopathological findings suggestive of bile duct involvement. Although AMAs were negative - a rare exception in PBC diagnosis - other features supported the diagnosis. For AIH, the patient met all three criteria: elevated ALT levels at 380 U/L (exceeding five times the ULN), significantly elevated serum IgG at 3700 mg/dL (above twice the ULN), and histological findings on liver biopsy consistent with chronic hepatitis. The biopsy revealed a dense portal lymphoplasmacytic infiltrate, moderate interface hepatitis, and mild lobular necroinflammation, characteristic of moderate-to-severe periportal or periseptal lymphocytic piecemeal necrosis. Together, these findings confirmed the diagnosis of AIH-PBC overlap syndrome, a rare and challenging condition to manage. In this case, the patient demonstrated a positive response to a combination therapy with ursodeoxycholic acid, prednisolone, and azathioprine, which aligns with the recommended approach for managing overlap syndrome to effectively reduce symptoms and improve biochemical markers [8].

## Conclusions

AIH-PBC overlap syndrome is a complex condition requiring a multidisciplinary approach for accurate diagnosis and effective management. Guided by the "Paris criteria," the diagnosis relies on elevated LFTs, positive autoimmune markers, and liver biopsy findings. Treatment with ursodeoxycholic acid, prednisolone, and azathioprine resulted in significant improvement. Regular monitoring of liver function, autoimmune markers, and potential complications is essential to achieving remission and optimizing long-term outcomes.
